# *Ras* Oncogene-Mediated Progressive Silencing of Extracellular Superoxide Dismutase in Tumorigenesis

**DOI:** 10.1155/2015/780409

**Published:** 2015-10-15

**Authors:** Francesca Cammarota, Gabriella de Vita, Marco Salvatore, Mikko O. Laukkanen

**Affiliations:** ^1^IRCCS SDN, 80143 Naples, Italy; ^2^Department of Molecular Medicine and Medical Biotechnologies, University of Naples Federico II, 80014 Naples, Italy

## Abstract

Extracellular superoxide dismutase (SOD3) is a secreted enzyme that uses superoxide anion as a substrate in a dismutase reaction that results in the formation of hydrogen peroxide. Both of these reactive oxygen species affect growth signaling in cells. Although SOD3 has growth-supporting characteristics, the expression of *SOD3* is downregulated in epithelial cancer cells. In the current work, we studied the mechanisms regulating *SOD3* expression *in vitro* using thyroid cell models representing different stages of thyroid cancer. We demonstrate that a low level of RAS activation increases *SOD3* mRNA synthesis that then gradually decreases with increasing levels of RAS activation and the decreasing degree of differentiation of the cancer cells. Our data indicate that *SOD3* regulation can be divided into two classes. The first class involves RAS–driven reversible regulation of *SOD3* expression that can be mediated by the following mechanisms: RAS GTPase regulatory genes that are responsible for *SOD3* self-regulation; RAS-stimulated p38 MAPK activation; and RAS-activated increased expression of the *mir21* microRNA, which inversely correlates with *sod3* mRNA expression. The second class involves permanent silencing of *SOD3* mediated by epigenetic DNA methylation in cells that represent more advanced cancers. Therefore, the work suggests that *SOD3* belongs to the group of *ras* oncogene-silenced genes.

## 1. Introduction

The regulation of* extracellular superoxide dismutase* (*SOD3*) gene expression has remained challenging. Temporal stimulation of SOD3 expression could benefit tissue healing after injury [1–6], while long-term inhibition of overproduction of the enzyme could prevent unwanted hyperplasia [7]. According to recent publications, SOD3 supports a number of growth-stimulating events in tumorigenesis. Moderate sustained overexpression of SOD3 induces nontransformed murine primary cell proliferation, causing immortalization and neoplastic transformation [7]. In aggressive anaplastic cancer cells, moderate sustained overexpression of SOD3 stimulates tumorigenesis and growth factor signal transduction [7, 8]. Moreover, SOD3 can control small GTPase activation and downstream signal transduction by modifying the expression of small GTPase regulatory genes [8, 9].

In tumorigenesis, genes that are expressed in normal cells but are disadvantageous for cancer development by inducing apoptosis [10], limiting cell proliferation [11], or correcting DNA damage [12] are downregulated through genetic events, such as deletions [13] and point mutations [14], epigenetic mechanisms, such as methylation and histone acetylation [15–17], and microRNA targeting affecting the gene transcription [18]. Intriguingly, SOD3, which has several growth-supporting characteristics, such as the ability to promote mitogenic and cell survival signaling, is frequently downregulated in advanced epithelial cancer cells. Thus, the role of SOD3 in tumorigenesis requires further study. To systematically characterize the concomitant mechanisms causing* SOD3* downregulation in epithelial cancer cells, we utilized rat and human thyroid cell models harboring different oncogenes and representing variable degrees of differentiation. Based on our observations,* sod3* mRNA expression is progressively altered corresponding to RAS GTPase activation and carcinogenesis, thus further strengthening our previous observations suggesting a close cooperative connection between SOD3 and RAS.

## 2. Methods

### 2.1. Cells

Rat thyroid PC Cl3, PC PTC1, PC E1A, and FRLT5 and the related cell clones V13, V21, and V27 stably transfected with an* H-RasV12* expression plasmid [19] were cultured in Ham's F12 medium Coon's modified (Sigma, St. Louis, MO, USA) supplemented with 5% calf serum (Life Technologies, Inc., Carlsbad, CA), penicillin (100 U/mL) (Sigma), and streptomycin (100 mg/L) (Sigma). PC Cl3 and FRLT5 cells (modeling normal thyroid cells) were additionally supplemented with 10 nM TSH, 10 nM hydrocortisone, 100 nM insulin, 5 mg/mL transferrin, 5 nM somatostatin, and 20 mg/mL glycyl-histidyl-lysine. Human thyroid NTHY-ori 3.1 (Nthy) and anaplastic thyroid cancer 8505c cells were grown in RPMI medium (Life Technologies, Inc.) supplemented with 10% fetal bovine serum (Sigma), penicillin (100 U/mL), and streptomycin (100 mg/L). Human papillary thyroid cancer TPC1 cells were grown in DMEM supplemented with 10% fetal bovine serum, penicillin (100 U/mL), and streptomycin (100 mg/L). HEK 293T cells were grown in DMEM supplemented with 10% fetal bovine serum, penicillin (100 U/mL), and streptomycin (100 mg/L). To study the regulation of* sod3* gene expression, the cells were supplemented with 10 *μ*M 5-azacytidine (Sigma), 100 nM TSA (Sigma), and 10 *μ*M SB202190 p38 MAPK inhibitor (Sigma) for 24 hours or with H_2_O_2_ (Sigma) for 6 hours.

The human* SOD3* expression vector (a gift from Professor Stefan L. Marklund, University of Umea, Sweden), rabbit* sod3* expression vector previously cloned in our group [20], or pcDNA3 control (Invitrogen, Paisley, UK) or* H-RasV12* plasmid was transfected with Fugene 6 (Promega, WI, USA) according to standard protocols.

### 2.2. Real-Time Reverse Transcription PCR

Cells were pelleted for RNA isolation using an RNeasy Mini Kit (Qiagen, Hilden, Germany) and cDNA synthesis using a QuantiTect Reverse Transcription Kit (Qiagen). The PCR reaction was performed using an iCycler (BioRad, Hercules, CA, USA) and SYBR Green PCR Master Mix (Applied Biosystems, Foster City, CA). The primers were designed with (http://www.ncbi.nlm.nih.gov/tools/primer-blast/). Human *β-actin* and rat *β-actin* were used to normalize human and rat templates, respectively. Relative fluorescence expression values were calculated as follows: ΔCt = Ct (gene of interest)-Ct (*β*-actin) ΔΔCt = ΔCt (treated)-ΔCt (control). Relative expression values = 2^(−ΔΔCt)^. The primers are listed in Table 1.

### 2.3. MicroRNA Isolation

MicroRNA was isolated using an miRNeasy Kit (Qiagen) for reverse transcription with a miScript RT II Kit (Qiagen). The* miR21* and* RNU5* miScript Primer Assays (Qiagen) were used for amplification and to normalize the* miR21* expression.

### 2.4. Western Blot

The cells were harvested in lysis buffer (50 mM HEPES, pH 7.5, 150 mM NaCl, 10% glycerol, 1% Triton X-100, 1 mM EGTA, 1.5 mM MgCl_2_, 10 mM NaF, 10 mM sodium pyrophosphate, 1 mM Na_3_VO_4_, 10 *μ*g aprotinin/mL, and 10 *μ*g leupeptin/mL), separated by sodium dodecyl sulfate–polyacrylamide gel electrophoresis, and transferred to Hybond C Extra nitrocellulose membranes (GE Healthcare, WI, USA). The following antibodies were used: pErk1/2, Erk1/2, p-p38 MAPK, and p38 MAPK (Cell Signaling Technology, MA, USA).

### 2.5. Statistical Analysis

The experiments were repeated at least three times. All of the results were expressed as the mean values ± SD. The *p* values (^*∗*^ *p* < 0.05,  ^*∗∗*^ *p* < 0.01, and ^*∗∗∗*^ *p* < 0.001) were determined using two-tailed independent sample *t*-tests.

## 3. Results

### 3.1. Thyroid Cell Models Representing Different Degrees of Thyroid Cancer

Thyroid stimulating hormone, hydrocortisone, insulin, transferrin, somatostatin, and glycyl-histidyl-lysine supplementation-dependent FRLT5 cells are a clonal cell line derived from rat normal thyroid FRLT cells by purification through successive isolation of colonies [21]. FRLT5 clones V13, K20, K2, V21, V39, and V27 were created by stable transfection with* H-RasV12* oncogene followed by clonal expansion of the cell lines derived from a single cell. The clone V13 harbors 1.4-fold, K20 3.1-fold, K2 6.3-fold, V21 10-fold, V39 35-fold, and V27 46-fold RAS activity relative to parental cells. This increased RAS activity drives the growth of the cells without hormone supplementation, thus modeling the hormone-independent growth characteristics of thyroid cancers [19]. Similar to FRLT5 cells, PC Cl3 cells represent rat normal thyroid cells and are dependent on hormone supplementation [22]. PC PTC1 and PC E1A clones are PC Cl3 derivatives that were transformed with* PTC1* and* E1A* oncogenes [23].* PTC1* is a papillary thyroid cancer- (PTC-) associated oncogene derived from a genomic rearrangement of the* RET* oncogene with the* CDC61/H4* gene [24]. The* E1A* oncogene shows structural and functional similarities to the* MYC* and* MYB* oncogenes [25] that mechanistically bypass the proliferative block of terminally differentiated cells by increasing the activity of cell cycle-related proteins [26].

To model normal human thyroid, we utilized SV40-immortalized Nthy cells [27], which are able to grow without hormone supplementation. TPC1 cells, which are spontaneously transformed thyroid cancer cells isolated from a patient, represent well-differentiated papillary thyroid cancer in this study. The 8505c cell line originates from a patient with undifferentiated highly aggressive anaplastic thyroid cancer harboring mutant p53 [28] and a BRAF V600E mutation, resulting in constitutively active MEK-ERK kinase signaling with consequent uncontrolled cell proliferation and tumorigenesis [29]. HEK 293T cells derived from human embryonic kidney cells by adenovirus 5 transduction were used in the study to corroborate observations from thyroid cell model systems because of their strong growth and untroublesome transfection characteristics.

### 3.2. RAS Activation Correlates with* sod3* and* mir21* Expression

**Figure 1:**
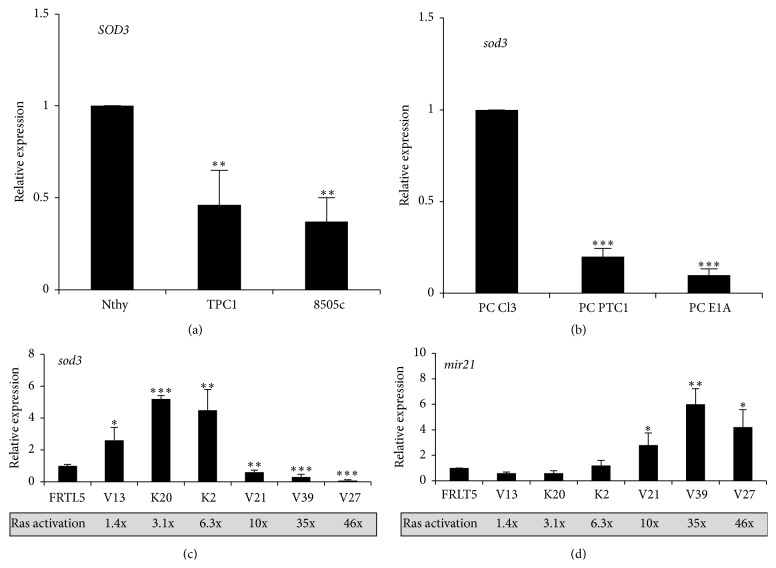
*SOD3* mRNA expression level in the cell model systems used in this study. (a)* SOD3* expression analysis in human papillary thyroid TPC1 and anaplastic thyroid 8505c cells showed significantly reduced mRNA expression compared to Nthy cells representing normal thyroid. (b) The* sod3* mRNA expression from rat thyroid PC Cl3 cells modeling normal thyroid, PC PTC1 transformed with the PTC1 oncogene, and PC E1A cells transformed with the E1A oncogene. The oncogenic transformation of PC Cl3 cells caused a downregulation of* sod3* mRNA. (c) The expression analysis of* sod3* from rat FRLT5 cell clones harboring 1.4-fold to 46-fold increased RAS activity compared to control FRLT5 cells suggested significantly increased* sod3* expression in the clones V13 (1.4-fold RAS activity), K20 (3.1-fold RAS activity), and K2 (6.3-fold RAS activity). A significant decrease in sod3 mRNA synthesis was observed in the clone V21 (10-fold RAS activity) that was further reduced in the clones V39 (35-fold RAS activity) and V27 (46-fold RAS activity). (d) The expression analysis of the microRNA* mir21* from FRLT5 cells. Compared to control FRLT5 cells, the expression levels of* mir21* were similar in the clones V13 (1.4-fold RAS activity), K20 (3.1-fold RAS activity), and K2 (6.3-fold RAS activity). A significant increase in expression was observed in the clones V21 (10-fold RAS activity), V39 (35-fold RAS activity), and V27 (46-fold RAS activity). The data are expressed as the mean ± SD. The *p* values are represented (^*∗*^ *p* < 0.05, ^*∗∗*^ *p* < 0.01, and ^*∗∗∗*^ *p* < 0.001).

To validate the cell models for the aims of the current study, we investigated the expression of* SOD3* mRNA.* SOD3* expression was gradually downregulated in papillary thyroid cancer TPC1 cells and anaplastic thyroid cancer 8505c cells compared to control Nthy cells modeling normal thyroid cells (Figure 1(a)), suggesting a correlation between the mRNA expression and the degree of differentiation of the cancer [7, 30]. We observed a similar downregulation of* sod3* mRNA synthesis in PC Cl3 cells transformed with other oncogenes (Figure 1(b)), confirming the oncogene-dependent regulation that has previously been observed [30]. According to a recent report, transfection of* H-RasV12*,* b-raf*,* mek*, and* erk* into cells stimulates* SOD3* production [9]. To investigate the mechanism by which increased activation of RAS controls* sod3* mRNA production, we utilized rat thyroid FRLT5 cell line clones stably transfected with varying amounts of the* H-RasV12* oncogene [19]. Increased RAS activation of 1.4-fold to 6.3-fold stimulated* sod3* expression, whereas 10-fold RAS activation caused a striking decrease in the mRNA production that was further enhanced at 35-fold and 46-fold RAS activation levels (Figure 1(c)).

A recent report has suggested that* mir21* targets the* SOD3* 3′UTR and results in RNAi-mediated silencing of the gene expression [31]. We assessed* mir21* expression in the FRLT5 and FRLT5-derived clones and observed increased microRNA production that correlated with increased RAS activity [19]. Low 1.4- to 6.3-fold increased RAS activation did not increase* mir21* production, whereas a significant strong induction was observed with 10-fold RAS activation (Figure 1(d)). These data corroborate the connection between* sod3* and* mir21* expression.

### 3.3. RAS-Induced p38 MAPK Phosphorylation Inhibits* sod3* mRNA Expression

A previous study has suggested that activation of p38 MAPK, one of the RAS downstream target cellular kinases, contributes to the regulation of* SOD3* production [32]. We therefore investigated the effect of the p38 MAPK inhibitor SB202190 on* SOD3* mRNA synthesis in the thyroid cell models.

**Figure 2:**
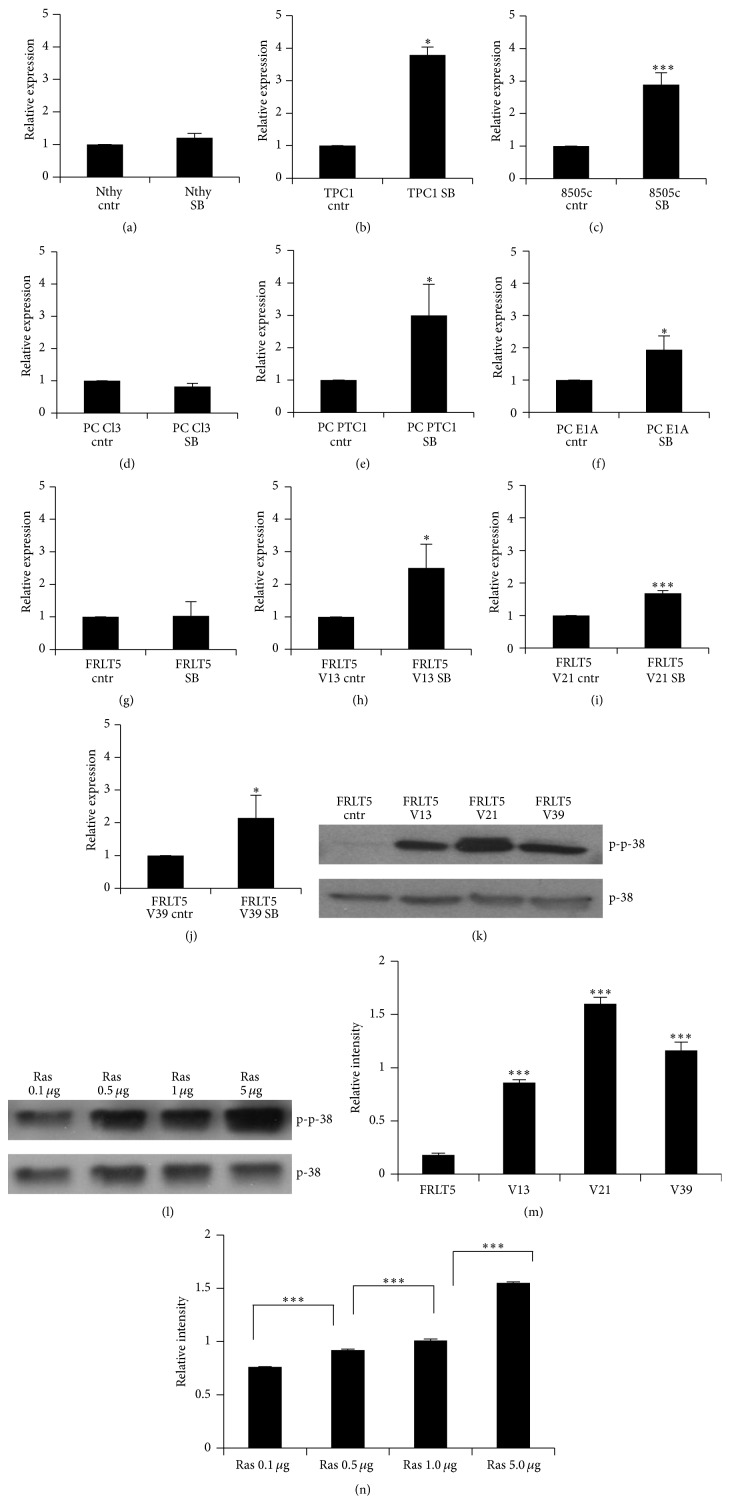
Effect of the p38 MAPK inhibitor SB202190 on* SOD3* mRNA expression. (a–c) The inhibition of p38 MAPK phosphorylation increased the* SOD3* expression in human TPC1 and 8505c cells, while SB202190 treatment did not alter the* SOD3* mRNA synthesis in Nthy cells modeling normal thyroid. (d–f) The inhibitor treatment increased* sod3* mRNA expression in PC Cl3-derived PC PTC1 and PC E1A cells, while the treatment had no effect on PC Cl3 control cells modeling normal thyroid. (g–j) FRLT5 cell clones V13, V21, and V39 stably transfected with RAS showed a significant increase in* sod3* mRNA expression after SB202190 treatment, whereas SB202190 had no effect on FRLT5 control cells, which is consistent with the results using the other cell models. (k, m) The Western blot analysis for p38 MAPK activation in FRLT5 control cells, clone V13, clone V21, and clone V39. The histogram (panel (m)) suggested significantly increased p38 MAPK phosphorylation in FRLT5-derived clones compared to control cells. Total p38 MAPK was used to normalize the phosphorylation level. (l, n) The transient transfection of an* H-RasV12* expression plasmid into HEK 293T cells showed a gradual increase in p38 MAPK phosphorylation that corresponded to the amount of plasmid transfected. Total p38 MAPK was used to normalize the phosphorylation level. The data are expressed as the mean ± SD. The *p* values are represented (^*∗*^ *p* < 0.05, ^*∗∗*^ *p* < 0.01, and ^*∗∗∗*^ *p* < 0.001).

Inhibition of the kinase had a minor impact on the* SOD3* gene expression in human Nthy cells (Figure 2(a)), whereas* SOD3* expression was significantly increased in human TPC1 papillary thyroid cancer cells and in human 8505c anaplastic thyroid cancer cells (Figures 2(b) and 2(c)). These data suggest that p38 MAPK downregulates* SOD3* mRNA synthesis in advanced thyroid cancer cells. The expression data from PC Cl3 cells, PC PTC1 cells transformed with the papillary thyroid cancer PTC oncogene, and PC E1A cells immortalized with the aggressive adenovirus E1A oncogene (Figures 2(d)–2(f)) treated with the p38 inhibitor indicated similar upregulation of* sod3* expression, suggesting oncogene-dependent silencing of* sod3* via p38 MAPK activation. The oncogene-derived effect on p38 MAPK activation and* sod3* downregulation was then studied in FRLT5 cells. In accord with the other cell models used, the kinase inhibitor minimally affected FRLT5 cells, whereas a significant increase in mRNA production was observed in FRLT5 clones V13, V21, and V39 harboring 1.4-fold, 10-fold, and 35-fold RAS activity (Figures 2(g)–2(j)). Thus, the data corroborate previous observations [32] suggesting that activation of RAS through p38 MAPK downregulates* sod3* expression, whereas inhibition of p38 MAPK phosphorylation increases* sod3* mRNA production.

We then analyzed the p38 MAPK phosphorylation status in FRLT5 cells and related clones. The analysis showed significantly increased phosphorylation levels in the clones V13, V21, and V39 corresponding to RAS activation, hence corroborating p38 MAPK inhibitor results and suggesting that the RAS-p38 MAPK signal transduction pathway controls* sod3* mRNA synthesis (Figures 2(k) and 2(m)). The involvement of the RAS-p38 MAPK signaling pathway was further confirmed by transient transfection of HEK 293T cells with increasing concentrations of the* H-RasV12* oncogene; the increase in* H-RasV12* levels correlated with increased p38 MAPK phosphorylation (Figures 2(l) and 2(n)).

### 3.4. Cancer-Induced Hypermethylation Downregulates* sod3* mRNA Expression

**Figure 3:**
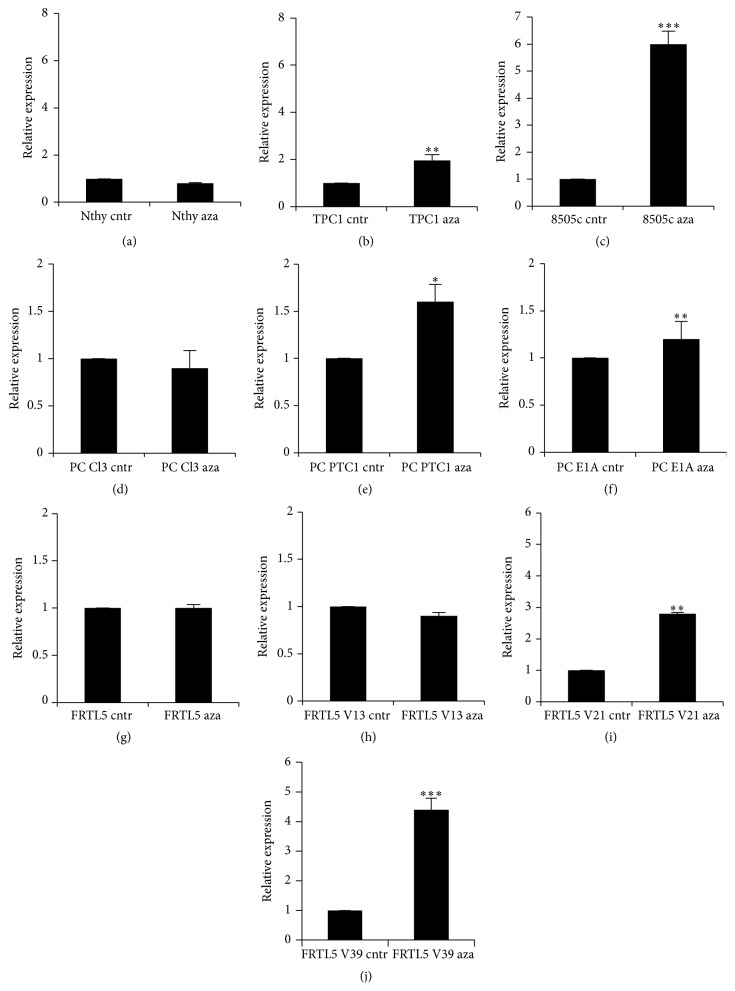
Effect of the demethylation reagent 5-azacytidine on* SOD3* mRNA expression. (a–c) Nthy cells modeling normal human thyroid cells showed similar* SOD3* mRNA expression in nontreated and treated control cells, whereas the mRNA production was significantly increased in 5-azacytidine-treated papillary TPC1 and anaplastic 8505c thyroid cancer cells. (d–f) Similar to the human cells, no alteration in* sod3* mRNA was observed in rat PC Cl3 cells modeling normal rat thyroid cells after hypomethylation treatment, whereas the treatment induced increased mRNA synthesis in PTC1 and E1A oncogene-transfected cells. (g–j) The hypomethylation treatment did not affect normal rat thyroid FRLT5 cells or FRLT5 clone V13 cells harboring low 1.4-fold RAS activity, whereas a significant increase in the* sod3* mRNA production was observed in clones V21 and V39 harboring 10-fold and 35-fold RAS activity, respectively. The data are expressed as the mean ± SD. The *p* values are represented (^*∗*^ *p* < 0.05, ^*∗∗*^ *p* < 0.01, and ^*∗∗∗*^ *p* < 0.001).

The coding sequence of the* sod3* gene is located in a hypermethylated CpG island [33], which suggests that methylation is involved in the regulation of the gene expression. Treatment with the hypomethylation agent 5-azacytidine did not affect* SOD3* mRNA expression in human Nthy cells, rat PC Cl3 cells, rat FRLT5 cells, or FRLT5 clone V13 containing a modest 1.4-fold increased RAS activation level (Figures 3(a), 3(d), 3(g), and 3(h)); these results are consistent with our previous data showing no impact of hypomethylation on* sod3* expression in aortic smooth muscle cells representing normal vascular tissue [33]. However, the 5-azacytidine treatment significantly increased* SOD3* expression in the human papillary thyroid cancer TPC1 and human anaplastic thyroid cancer 8505c cells (Figures 3(b) and 3(c)). Similarly, rat thyroid PC PTC1 and PC E1A cells (Figures 3(e) and 3(f)) and FRLT5 cell clones V21 and V39 with 10-fold and 35-fold increased RAS activation, respectively, demonstrated increased* sod3* mRNA expression (Figures 3(i) and 3(j)). The results thus suggest that methylation-mediated* SOD3* downregulation in cancer corresponds to the degree of differentiation of the cancer and to the activation level of the RAS small GTPase.

### 3.5. Histone Acetylation Affects* sod3* mRNA Expression in Cells That Model Normal Thyroid Tissue

**Figure 4:**
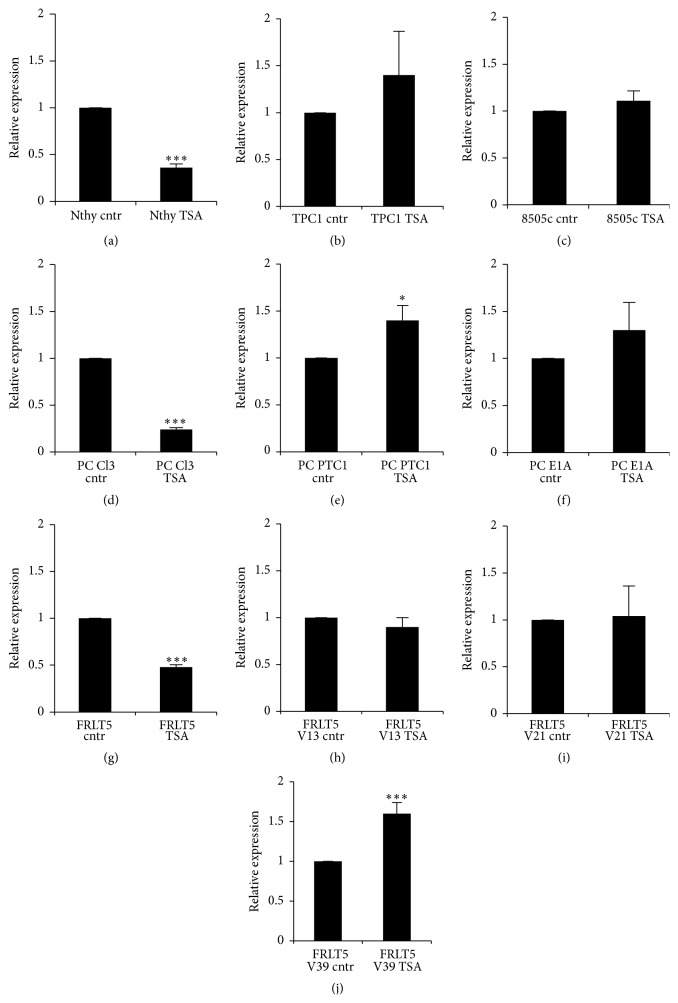
Effect of the histone deacetylation inhibitor trichostatin A (TSA) on* SOD3* mRNA expression. (a–c) Human thyroid cells showed reduced* SOD3* mRNA production in Nthy cells modeling normal thyroid cells and no alteration in papillary thyroid cancer TPC1 or anaplastic thyroid cancer 8505c cells. (d–f) The expression analysis from PC Cl3 cells supported the data obtained from the human cells;* sod3* expression was significantly reduced in control PC Cl3 cells and not altered in E1A transfected cells. (g–j) TSA treatment led to decreased* sod3* expression in FRLT5 cells modeling normal thyroid but did not affect expression in clone V13 or V21 stably transfected with the* H-RasV12* oncogene. TSA treatment increased* sod3 *mRNA synthesis only in PC PTC1 and FRLT5 V39 cells. The data are expressed as the mean ± SD. The *p* values are represented (^*∗*^ *p* < 0.05, ^*∗∗*^ *p* < 0.01, and ^*∗∗∗*^ *p* < 0.001).

Histone deacetylases regulate the expression of genes involved in the initiation of tumorigenesis. Deacetylation creates a tight nonpermissive chromatin conformation that inhibits transcription. The histone deacetylation inhibitor trichostatin A (TSA) interferes with the histone deacetylase function of removing the acetyl groups from the histones, therefore increasing the histone acetylation level and resulting in the formation of an open euchromatin structure. Treatment with TSA significantly (*p* < 0.001) decreased* sod3* mRNA expression in all of the thyroid cells that model normal thyroid (Figures 4(a), 4(d), and 4(g)), while only a tendency towards increased expression was observed in transformed and cancer cells (Figure 4).

### 3.6. Self-Regulation of* sod3* Expression Is Controlled by RAS Small GTPase Regulatory Genes

**Figure 5:**
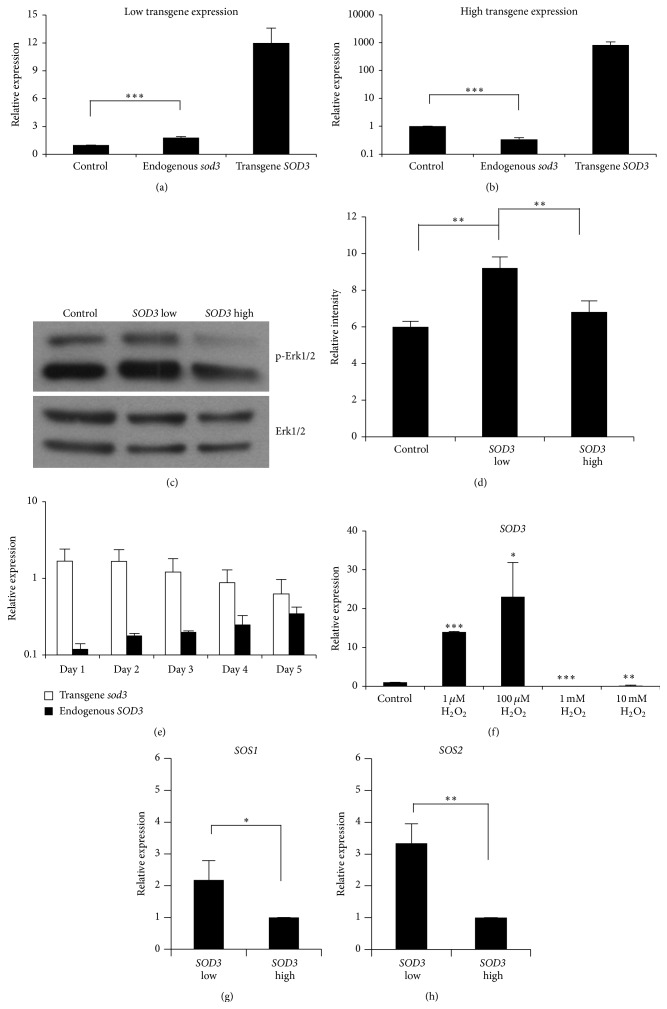
Self-regulation of* SOD3* expression is mediated by GEF small GTPase regulatory proteins. (a-b) The analysis of the effects of low and high* SOD3* transgene transfection on endogenous* sod3* mRNA synthesis in rat PC E1A cells. Transfection of low amounts of human* SOD3* into PC E1A cells significantly increased the endogenous rat* sod3* expression, whereas high amounts of human* SOD3* levels in PC E1A cells significantly reduced the endogenous rat* sod3* mRNA synthesis. (c-d) Western blot analysis of ERK1/2 phosphorylation in PC E1A cells suggested greater MAPK ERK1/2 activation at low* SOD3* transgene expression levels compared to high transgene levels. (e) Comparison of rabbit transgene* sod3* expression to human endogenous* SOD3* mRNA synthesis in HEK 293T cells. The expression analysis performed on five consecutive days demonstrated a gradual decrease in the transgene expression that correlated with a gradual increase in the endogenous* SOD3* mRNA synthesis. (f) The effect of the H_2_O_2_ concentration on* SOD3* mRNA expression. HEK 293T cells were treated with different concentrations of H_2_O_2_. Low H_2_O_2_ concentrations caused increased* SOD3* mRNA synthesis, and high H_2_O_2_ levels caused decreased* SOD3* expression. (g) The effects of low and high* SOD3* transgene expression on* SOS1* and* SOS2* GEF mRNA expression in 8505c cells. A low level of transgene expression caused an increase in* SOS1* and* SOS2* mRNA production, whereas high transgene* SOD3* levels decreased the expression of both GEFs. The data are expressed as the mean ± SD. The *p* values are represented (^*∗*^ *p* < 0.05, ^*∗∗*^ *p* < 0.01, and ^*∗∗∗*^ *p* < 0.001).

Our previous results have demonstrated SOD3-driven increased RAS GTP loading and RAS-ERK1/2-derived* sod3* mRNA expression, therefore suggesting a self-regulatory loop for SOD3 [9]. This observation was further corroborated by data indicating that SOD3 causes increased growth signaling at moderate expression levels [7] and inhibits activation of the small GTPases RAS, RAC, CDC42, and RHO at high enzyme levels [8]. To evaluate the concentration-dependent self-regulation of* SOD3*, we transfected 0.25 *μ*g and 10 *μ*g of human* SOD3* into the rat thyroid PC E1A cell line, which expresses low levels of endogenous* sod3* mRNA. Transfection with low amounts of human* SOD3* significantly increased the rat* sod3* expression (Figure 5(a)), whereas transfection with high amounts of* SOD3* had the opposite effect, significantly decreasing rat* sod3* mRNA synthesis (Figure 5(b)). These data were supported by Western blot analysis suggesting a* SOD3* dose-dependent activation of ERK1/2 kinase in PC E1A cells (Figures 5(c) and 5(d)). We have previously demonstrated that ERK1/2 stimulates endogenous* SOD3* expression [9]. To corroborate the dose-dependent self-regulation, we transfected rabbit* sod3* into human HEK 293T cells and analyzed species-specific mRNA production on consecutive days. While the rabbit* sod3* transgene expression decreased with increasing time from transfection, the endogenous human* SOD3* mRNA production increased to maintain the total rabbit/human* sod3* mRNA expression at similar levels on different days (Figure 5(e)). Because the only known physiological function of SOD3 is to dismutase superoxide to hydrogen peroxide, we investigated the effect of hydrogen peroxide on* SOD3* mRNA synthesis. Treatment with low levels of H_2_O_2_ increased the endogenous* SOD3* expression, while treatment with high levels of H_2_O_2_ (1 mM and 10 mM) significantly decreased the mRNA production (Figure 5(f)); these data support the dose-dependent self-regulation data.

We have recently demonstrated that SOD3 activates a number of tyrosine kinase receptors, cell membrane-associated nontyrosine kinases, and cellular kinases [8, 9, 34]. Based on our recent data, SOD3 regulates mitogen signal transduction by controlling the expression of small GTPase regulatory genes, including guanine nucleotide exchange factors (GEF), GTPase activating proteins (GAP), and guanosine nucleotide disassociation inhibitors (GDI), thus regulating the activation level of RAS [8]. In the present work, we investigated in 8505c cells the effects of low and high* SOD3* expression levels on the guanine nucleotide exchange factor genes* SOS1* and* SOS2*, which are responsible for RAS GTP loading. The data suggested that* SOS1* and* SOS2* mRNA expression is higher in cells that harbor low* SOD3* mRNA content compared to cells with high* SOD3* mRNA synthesis (Figures 5(g) and 5(h)). The data are in agreement with our previous results, in which we have shown increased growth and increased* RhoGEF16* and* RalGEF RGL1* expression with moderately increased* SOD3* mRNA levels, whereas high* SOD3* expression led to decreased growth and* RhoGEF16* and* RalGEF RGL1* expression [8]. Hence, the results suggest that SOD3 self-regulation is coordinated through guanine nucleotide exchange factors that are responsible for RAS activation.

## 4. Discussion

The signal transduction-related events that stimulate or inhibit* SOD3* expression have previously been described and suggest the involvement of cytokines [35, 36], epigenetic regulation [37, 38], MAP kinases [9, 32], and the microRNA* mir21* [31]. While the expression of the SOD3 enzyme is relatively stable in normal tissues, a significant increase in* sod3* mRNA levels has been observed in rat thyroid benign tumors [30] with sequential downregulation of expression in carcinogenesis that corresponds to the degree of differentiation of the cells [30]. To characterize the cellular mechanisms causing this downregulation, we investigated the influence of the known regulatory factors in well-described thyroid cancer models systems in the current study.

Notably, the expression of* SOD3* correlated with the activation level of RAS (Figure 1), suggesting that* SOD3* is a* ras* oncogene-downregulated gene. Based on the data, the regulation of* SOD3* mRNA expression in the thyroid cell models can be divided into immediate regulatory events, which are reversible and depend on the RAS activity, and late regulatory events, which cause long-term permanent silencing of the gene. The self-regulation mediated through RAS small GTPase regulatory genes may represent the fastest means of regulating* SOD3* expression, which is required to maintain stable expression of the mRNA production and the redox balance in tissues. The guanine nucleotide exchange factors (GEF) small GTPase regulatory genes contribute to RAS activation by active removal of GDP that is then replaced by GTP with consequent immediate response in the downstream signal transduction. The current data suggest that even minor alterations in* SOD3* expression significantly affect* SOS1* and* SOS2 GEF* expression via a feedback loop, hence enabling response to sudden changes in signaling and fine adjustments of SOD3 production.

Recent papers have suggested that induction of* mir21* transcription occurs through RAS-AP1 signaling [39] in a dose-dependent manner that correlates with RAS activity. While low RAS activity showed a minor impact on* mir21* production, a striking increase in expression in cells with high RAS activity [19] was observed; these results are in accord with the current data. Thus, the microRNA* mir21* represents another immediate reversible regulatory step; however, this mechanism of regulation might require greater changes in RAS activation than GEF–mediated* SOD3* regulation. The expression of* mir21*, which binds to the 3′UTR region of* SOD3* mRNA [31], showed an inverse correlation with* sod3* mRNA expression (Figures 1(c) and 1(d)), suggesting a sudden increase in* mir21* and decrease in* sod3* expression at 10-fold RAS activity. Therefore, the data suggest the importance of 10-fold RAS activity for cellular redox balance and* mir21* target gene silencing. The microRNA* mir21* is a well-characterized oncomir that is upregulated by RAS in the early stages of tumorigenesis. A recent paper has suggested that knockdown of* mir21* in cells transplanted into mice significantly reduces tumorigenesis [40], underlining the importance of this microRNA in cancer development.

The third reversible RAS-dependent regulation step occurs through p38 MAPK activation. The ability of p38 MAPK to regulate* SOD3* mRNA expression is noteworthy because the mitogen signaling cascade BRAF, MEK1/2, and ERK1/2 are primarily responsible for the stimulation of* SOD3* synthesis. Although MAPK kinases follow specific signal transduction pathways to phosphorylate their cognate MAPKs (e.g., MEK1/2 activates ERK1/2 but not JNK), p38 MAPK displays selectivity between cognate and noncognate docking sites that might explain the interference between two signal transduction pathways [41]. However, we cannot exclude the possibility that the p38 MAPK inhibitor SB202190 is not specific for p38 or that negative feedback could activate alternative signal transduction pathways [42].

DNA methylation and histone acetylation represent more permanent DNA regulatory mechanisms compared to regulation controlled by GEFs,* mir21*, and p38 MAPK activation. DNA methylation, a stable modification that is often provoked by RAS, controls the expression of a number of genes through epigenetic mechanisms that recruit different transcriptional repressors in an orderly fashion. A recent publication has described step-by-step methylation silencing of the* fas* gene, suggesting coordinated action of 28 RAS-related epigenetic silencing effectors [17] that employ a well-organized signal transduction pathway. The silencing of RAS downstream genes is initiated by recruitment of PDK1, MAPK1, and S100Z as the first step, followed by 14 other steps that eventually culminate in DNMT1 activation and consequent DNA methylation [43]. Although methylation changes are observed even in precancerous stages with chronic inflammation, the current data suggest the most prominent role for methylation-mediated* SOD3* silencing in the presence of high RAS activity in advanced well-differentiated and poorly differentiated aggressive cancers. The first, the second, and the third exon of human* SOD3* locate inside or at close proximity of CpG islands and repetitive elements, such as Alu and LINE 2B retrotransposons [33]. Although* SOD3* promoter region is incompletely analyzed, it has been demonstrated that CpG methylation downregulates Sp1/Sp3 transcription factor binding to putative promoter region inhibiting* SOD3* promoter activity and mRNA synthesis* in vitro* in human A549 adenocarcinoma lung alveolar basal epithelial cells [37] thus being in line with the current data.

The transcriptional silencing of genes caused by DNA methylation is further reinforced by histone deacetylase (HDAC) activity, which inhibits histone acetylation and causes the formation of closed chromatin structure and reduced DNA transcriptional activity. CpG dimer methylation-associated proteins, such as the transcriptional repressor MeCP2, attract multiprotein complexes that contain corepressor histone deacetylases [44], which then inhibit the expression of genes controlling the cell cycle, such as* p21*, and promote uncontrolled growth and carcinogenesis [45]. The inhibition of HDAC function by TSA treatment had only a minor effect on* sod3* expression in cancer cells or FRLT5 cells harboring high RAS activity (Figure 4). Instead, HDAC inhibition significantly decreased* sod3* mRNA production in cells that modeled normal rat thyroid. In normal thyroid, thyroid stimulating hormone (TSH) production is linked to histone acetylation; TSA treatment inhibits TSH promoter activity and therefore TSH production [46]. We have previously demonstrated that hormonal starvation reduces* sod3* expression in thyroid cells, while TSH treatment stimulates* sod3* production that is mediated by the cAMP-PKA and PLC-Ca^2+^ signal transduction pathways and affects normal thyroid cell proliferation [30]. The TSA-mediated reduction in* SOD3* expression in human Nthy cells, which grow without hormone supplementation, suggests other TSH-independent mechanisms for gene regulation. However, the differences between human and rat cells could be caused by species-specific signal transduction [47], although indirect gene regulatory systems might also be involved.

In conclusion, the regulation of* SOD3* expression in carcinogenesis involves* ras* oncogene-driven sequential events consisting of both reversible mechanisms that correlate with the RAS activation levels and more stable epigenetic events. Importantly, the use of multiple cell lines harboring different oncogenes and representing varying degrees of transformation in different species suggests that the regulation of* SOD3* gene expression is not cell line-specific. Our current results thus suggest that* SOD3* is a* ras* oncogene-downregulated gene.

## Figures and Tables

**Table 1:** PCR primer sequences used in the study.

Human SOD3 forward	cttcgcctctgctgaagtct
Human SOD3 reverse	gggtgtttcggtacaaatgg
Rat sod3 forward	gacctggagatctggatgga
Rat sod3 reverse	gtggttggaggtgttctgct
^*∗*^Rabbit sod3 forward	gttgcgtgagcggaaaga
Rabbit sod3 reverse	gtgagcgcctgccagatctc
Human SOS1 forward	cacctcctcctcaaacacct
Human SOS1 reverse	gtgtgtgtgctcccttttgt
Human SOS2 forward	ttttgaagaacgggtggcag
Human SOS2 reverse	ttttcctttcctgcagtgcc
Human *β*-actin forward	tgcgtgacattaaggagaag
Human *β*-actin reverse	gctcgtagctcttctcca
Rat *β*-actin forward	tcgtgcgtgacattaaggag
Rat *β*-actin reverse	gtcaggcagctcgtagctct

^*∗*^Primer designed against EF1alfa promoter.
